# A Conserved Behavioral State Barrier Impedes Transitions between Anesthetic-Induced Unconsciousness and Wakefulness: Evidence for Neural Inertia

**DOI:** 10.1371/journal.pone.0011903

**Published:** 2010-07-30

**Authors:** Eliot B. Friedman, Yi Sun, Jason T. Moore, Hsiao-Tung Hung, Qing Cheng Meng, Priyan Perera, William J. Joiner, Steven A. Thomas, Roderic G. Eckenhoff, Amita Sehgal, Max B. Kelz

**Affiliations:** 1 Department of Medicine, University of Pennsylvania School of Medicine, Philadelphia, Pennsylvania, United States of America; 2 Department of Anesthesiology and Critical Care, University of Pennsylvania School of Medicine, Philadelphia, Pennsylvania, United States of America; 3 Howard Hughes Medical Institute, Philadelphia, Pennsylvania, United States of America; 4 Department of Pharmacology, University of Pennsylvania School of Medicine, Philadelphia, Pennsylvania, United States of America; 5 Mahoney Institute for Neurosciences, University of Pennsylvania, Philadelphia, Pennsylvania, United States of America; 6 Center for Sleep and Respiratory Neurobiology, University of Pennsylvania School of Medicine, Philadelphia, Pennsylvania, United States of America; 7 Institute for Translational Medicine and Therapeutics, University of Pennsylvania School of Medicine, Philadelphia, Pennsylvania, United States of America; Queensland Brain Institute, Australia

## Abstract

One major unanswered question in neuroscience is how the brain transitions between conscious and unconscious states. General anesthetics offer a controllable means to study these transitions. Induction of anesthesia is commonly attributed to drug-induced global modulation of neuronal function, while emergence from anesthesia has been thought to occur passively, paralleling elimination of the anesthetic from its sites in the central nervous system (CNS). If this were true, then CNS anesthetic concentrations on induction and emergence would be indistinguishable. By generating anesthetic dose-response data in both insects and mammals, we demonstrate that the forward and reverse paths through which anesthetic-induced unconsciousness arises and dissipates are not identical. Instead they exhibit hysteresis that is not fully explained by pharmacokinetics as previously thought. Single gene mutations that affect sleep-wake states are shown to collapse or widen anesthetic hysteresis without obvious confounding effects on volatile anesthetic uptake, distribution, or metabolism. We propose a fundamental and biologically conserved concept of neural inertia, a tendency of the CNS to resist behavioral state transitions between conscious and unconscious states. We demonstrate that such a barrier separates wakeful and anesthetized states for multiple anesthetics in both flies and mice, and argue that it contributes to the hysteresis observed when the brain transitions between conscious and unconscious states.

## Introduction

Alternating activity in neuronal networks is responsible for the daily fluctuation between states of conscious wakefulness and the unconsciousness associated with natural sleep [1]. As with wake and sleep, consciousness and anesthetic-induced unconsciousness are bistable, as subjects exist in only one of the mutually exclusive states at a time [2], [3]. Mathematical models of bistable systems predict the existence of hysteresis between the stable states [3]. Hysteresis is defined by the existence of distinct forward and reverse paths between the two stable states. The area enclosed by the hysteresis loop can be measured and often carries physical significance, for example the work, heat, or energy lost, which are respectively determined by integrating area under the pulmonary pressure-volume, force-length, or magnetization-magnetic field strength hysteresis loops [4], [5], [6].

**Figure 1:**
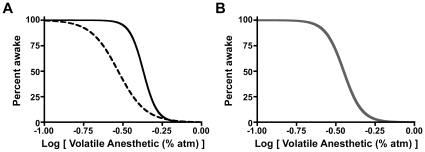
Schematic Data Demonstrating Path-Dependent and Path-Independent State Transitions. (**A**) Hysteresis defines path-dependent processes. The solid black curve represents a population of individuals entering the state of unconsciousness as a function of anesthetic dose. The dashed black curve represents the same population returning to a state of wakefulness as a function of anesthetic dose. (**B**) In the absence of hysteresis, the forward and reverse paths are superimposed (thick gray curve). Modeling studies of anesthetic-induced unconsciousness often collapse the hysteresis into a single curve to create path-independence. Experimental determination of arousal state at the steady-state anesthetic dose(s) half way between the top and bottom asymptotes, the EC_50_, can easily distinguish path-dependent from path-independent processes. In the former, the EC_50_ for induction and emergence differ significantly, whereas in the latter they are statistically indistinguishable.

Hysteresis in anesthetic-induced unconsciousness has been theorized using mathematical models of the transitions to and from anesthetic-induced unconsciousness [7]. At the central nervous system level, anesthetics exert their hypnotic effects in part by interacting with neuronal circuits that participate in the control of sleep and wakefulness [8], [9], [10], [11]. Hence, one might expect anesthetics to influence the hysteresis predicted to exist in the flip-flop switch that regulates states of natural sleep and wakefulness [2], [3]. However, rather than embracing hysteretic models of anesthetic-induced disruption and subsequent restoration of consciousness, existing models commonly collapse the hysteresis loop (Figure 1) into a single sigmoidal curve using pharmacokinetics to invoke an idealized anesthetic concentration at its effect site [12], [13], [14]. Nonetheless, evidence persists to suggest path-dependence of the transitions to and from unconscious states [14].

In this manuscript, we demonstrate that hysteresis in the onset and offset of anesthetic-induced unconsciousness cannot be fully explained by pharmacokinetics. We postulate that the area under the anesthetic dose-response hysteresis loop serves as a useful metric to unmask an intrinsic property of the central nervous system, namely the inherent resistance to changes in arousal state, which we term neural inertia. In two different species, we also present evidence that the barrier separating conscious and unconscious states is amenable to genetic and pharmacologic manipulation and is modulated by specific arousal-promoting mechanisms. The finding that specific gene products can affect the barrier size and thus the magnitude of hysteresis further excludes a pharmacokinetic explanation. In addition, genes and circuits related to arousal and sleep are implicated in the control of neural inertia. Thus, it is likely that understanding the mechanisms underlying neural inertia will provide insights into the regulation of sleep as well as states in which return of consciousness is pathologically impaired [15].

## Results

### Wild-type mice exposed to volatile anesthetics exhibit hysteresis between induction and emergence

**Figure 2:**
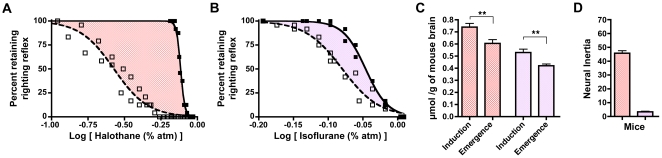
Neural Inertia In Wild Type Mice. Within the shaded area, subjects will be awake or anesthetized depending upon their previous state of arousal. This area represents a resistance to change in arousal state and graphically depicts neural inertia. (**A**) Halothane dose-response curve in wild-type mice for induction and emergence. (**B**) Isoflurane dose-response in wild-type mice. (**C**) One-way ANOVA with post-test Bonferroni multiple comparisons correction indicates that the residual volatile anesthetic in mouse brain at the corresponding EC_50_ for emergence is always significantly less than that at the EC_50_ for induction. ^**^p<0.01. (**D**) Neural inertia in wild-type mice exposed to halothane (red) or isoflurane (purple). X-axis in A and B corresponds to the log of the inhaled anesthetic concentration. All neural inertia bar graphs carry units of log[Anesthetic (%atm)]. Filled squares and solid curve denote induction. Open squares and dashed curve denote emergence.

By definition, anesthetic *induction* in mice occurs at the drug concentration at which the righting reflex is lost, whereas *emergence* occurs at the concentration at which the righting reflex returns. The EC_50_ for induction of halothane is more than 2.5 times that of the EC_50_ for emergence (Figure 2A, Table 1). Though the difference is smaller in magnitude, the EC_50_ for induction of isoflurane in mice is also significantly greater than the EC_50_ for emergence (Figure 2B, Table 1). To rule out an exclusive pharmacokinetic explanation, we determined the concentration of the anesthetic gases in brain at induction and emergence. Indeed, brain halothane and isoflurane concentrations at the EC_50_ for induction are always significantly greater than at emergence (Figure 2C). Because the central anesthetic concentration at the EC_50_ for induction exceeds that for the EC_50_ at emergence, anesthetic induction and emergence display path dependence (Figure 1). Moreover, for both anesthetics, the Hill slopes are also significantly greater for induction than emergence (Figure 2, Table 1). Significant differences in the EC_50_s and Hill slopes are consistent with the notion that emergence from anesthesia is not the mirror opposite process of induction.

**Table 1:** Best-fit parameters for volatile anesthetic studies in wild type mice and flies.

	Mouse				Fly			
	Halothane		Isoflurane		Halothane		Isoflurane	
	Induction	Emergence	Induction	Emergence	Induction	Emergence	Induction	Emergence
EC_50_ (%atm)	0.77	0.27	0.90	0.83	0.52	0.22	0.43	0.29
95% C.I. (%atm)	0.76–0.77	0.25–0.28	0.89–0.90	0.82–0.84	0.50–0.54	0.19–0.26	0.41–0.44	0.28–0.31
Hill slope	−30.9	−3.7	−26.8	−16.3	−11.8	−3.4	−9.0	−5.1
95% C.I. (%atm)	−38.2 to −23.7	−4.3 to −3.1	−30.4 to −23.2	−19.0 to −13.6	−15.5 to −8.1	−5.2 to −1.6	−11.5 to −6.6	−6.3 to −3.9
Top	100	100	100	100	99	63	98	69
95% C.I. Top	N.A.	N.A.	N.A.	N.A.	89–100	56–71	92–100	62–76

Top is set to 100% in mice.

### Neural inertia provides a quantitative measurement of the barrier separating states of anesthesia and wakefulness

While significant differences exist in the concentration of anesthetic at which half of the test population enter and exit the state of anesthesia (induction EC_50_>emergence EC_50_), a more comprehensive description is shown graphically by the shaded area bracketed between the solid induction and dashed emergence curves (Figure 2A–B). Integration of both curves over the range of the induction curve's EC_1_ through the emergence curve's EC_99_ (Figure S1, Appendix S1) yields an area that is a quantitative measure of resistance to transitions between arousal states, which we define as neural inertia (Figure 2D).

### Wild-type *Drosophila* exhibit hysteresis in their behavioral state transitions

**Figure 3:**
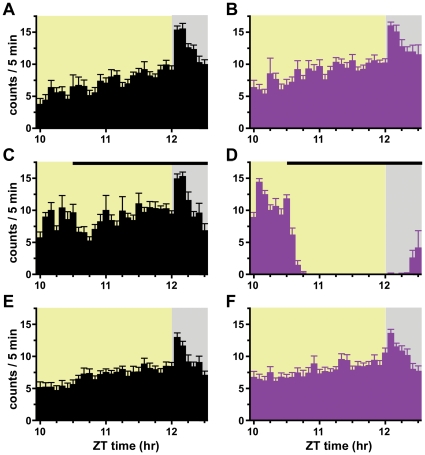
*Drosophila* Actograms of Locomotor Behavior. Wild-type *Iso31* flies entrained to a 12∶12 hour light (yellow)∶dark (gray) schedule display evening peak activity in pressurized air control (black) and isoflurane (purple) groups. One day prior to gas exposure, activity patterns are similar in the (**A**) air control group and (**B**) isoflurane group. On the experimental day, the duration of exposure to pressurized air with or without anesthetic gas is indicated by the thin horizontal black bar. (**C**) Activity pattern remains unchanged in the air control group. (**D**) Induction of anesthesia is marked by an abrupt cessation of activity in the isoflurane group, while emergence is marked by the resumption of activity. On the day following gas exposure, normal evening activity patterns are observed for flies previously exposed to (**E**) pressurized air without any anesthetic and (**F**) pressurized air containing isoflurane.

To determine if barriers impeding state transitions are conserved across evolution, we examined the effects of anesthetics on *Drosophila*, which have proven to be a useful model organism for studying anesthetic mechanisms [16], [17], [18], [19]. We developed a novel, high throughput assay to measure induction and emergence from anesthesia in flies. Anesthetic responsiveness in this assay is determined during the evening activity peak, when flies demonstrate consolidated wakefulness (Figure 3A–B) [20], [21]. On the experimental day, flies are exposed to stepwise increasing and then decreasing concentrations of an anesthetic in air. Control flies are exposed to an identical flow of air alone. Activity ceases in the population exposed to anesthetic and then gradually resumes upon emergence (Figure 3C–D). On the subsequent day, there are no gross differences in activity between flies previously exposed to an inhaled anesthetic or to air (Figure 3E–F).

**Figure 4:**
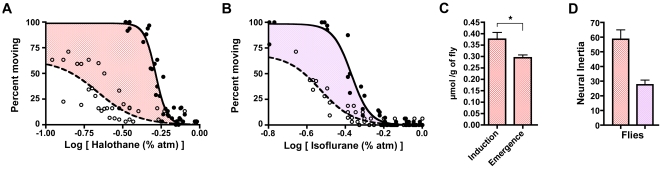
Neural Inertia In Wild Type *Drosophila*. (**A**) Halothane dose-response in wild-type *Iso31* flies. (**B**) Isoflurane dose-response in wild-type *Iso31* flies. (**C**) Unpaired t-test demonstrates that the residual halothane in a population of whole flies obtained at the corresponding EC_50_ for emergence is likewise significantly less than that at the EC_50_ for induction. (**D**) Neural inertia of wild type *Iso31* flies. X-axis in A and B corresponds to the log of the inhaled anesthetic concentration. Halothane is shown in red shading while isoflurane is shown in purple. Induction denoted by filled circles and solid curves, emergence by open circles and dashed curves. ^*^p<0.05.

Anesthetic *induction* in our assay is defined as the lowest concentration at which movement ceases for five or more minutes, whereas *emergence* is defined as the highest concentration at which movement resumes (Figure 4A–B). A five-minute immobility bout was chosen based upon definitions of *Drosophila* sleep [20], [21]. With these definitions, the EC_50_ for induction of halothane anesthesia in *Iso31* wild-type flies is more than 2.5 times that for emergence (Figure 4A, Table 1). Similarly, the EC_50_ for induction of isoflurane anesthesia in *Iso31* flies is significantly greater than that of emergence (Figure 4B, Table 1). Once again, Hill slopes for induction are significantly greater than for emergence (Table 1). The fly was chosen as a model organism in part because the lower diffusion barriers (as compared to a mammal) and hence faster equilibration renders a pharmacokinetic explanation for hysteresis unlikely. Nevertheless, were a pharmacokinetic confound present in the fly, it would be most obvious for the most lipid soluble compound, halothane. Therefore, we measured whole fly halothane concentration at the EC_50_ for induction and emergence and confirmed a significantly lower amount of halothane present at emergence (Figure 4C), further refuting an exclusively pharmacokinetic explanation. Experiments performed in a second strain of wild-type *RC1* flies, which carry the wild type *w* allele, yield similar data to the *Iso31* strain, which carry the *w^1118^* allele previously shown to influence anesthetic sensitivity [22], for both halothane and isoflurane (Figure S2).

### Barrier separating an anesthetized state from wakefulness can be manipulated genetically and pharmacologically in mice

**Figure 5:**
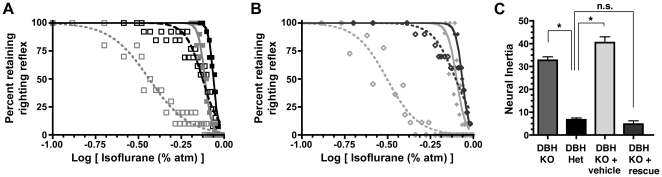
Neural Inertia May Be Modified Both Genetically As Well As Pharmacologically. (**A**) Mice deficient for the enzyme dopamine ß-hydroxylase (light gray squares) display hypersensitivity to induction (solid symbols, solid curve) of isoflurane anesthesia but a more significant phenotype of delayed emergence (open gray squares, dashed curve) leading to a profound increase in neural inertia relative to their sibling controls (black squares). (**B**) CNS-specific rescue of adrenergic signaling in these *Dbh* KO mice restores normal induction (solid black diamonds) and emergence (open black diamonds) with respect to vehicle-treated *Dbh* KOs (shown in light gray). (**C**) One-way ANOVA with post-test Bonferroni multiple comparisons correction indicates that neural inertia in *Dbh* KO mice is normalized only by CNS-specific rescue. X-axis in A and B corresponds to the log of the inhaled anesthetic concentration. ^*^p<0.05.

Based upon pharmacologic and lesion studies that impair adrenergic signaling and modulate supra-hypnotic anesthetic endpoints [23], [24], [25], we considered the adrenergic system as a candidate that might affect neural inertia. Therefore, we tested dopamine ß-hydroxylase (*Dbh*) null mice, which are devoid of norepinephrine and epinephrine, and compared them with *Dbh* heterozygous sibling controls shown previously to have normal catecholamine levels [26]. As predicted from previous studies [23], [24], [25], *Dbh* null mice exhibit hypersensitivity to induction by isoflurane. However, their most striking phenotype is a dramatic increase in neural inertia, due to a profoundly altered threshold for emergence (Figure 5A).

L-DOPS is a synthetic amino acid that can be converted to norepinephrine by aromatic L-amino acid decarboxylase in *Dbh* null mice. By pairing L-DOPS with a peripheral aromatic L-amino acid decarboxylase inhibitor, we achieve a CNS-specific acute rescue of adrenergic signaling in *Dbh* null mice while leaving all other peripheral tissues devoid of both epinephrine and norepinephrine [26]. Such CNS-specific rescue restores neural inertia to control levels by normalizing the EC_50_ and Hill slopes for induction and emergence. Conversely, injection of a vehicle control into *Dbh* null mice is ineffective (Figure 5B–C, Table 2). Together these experiments indicate that norepinephrine acts in the CNS to overcome the barrier opposing anesthetic emergence.

**Table 2:** Best fit parameters for isoflurane studies in *Dbh* null and heterozygous control mice.

	*Dbh* hets		*Dbh* KO		*Dbh* KO + rescue		*Dbh* KO + vehicle	
	Induction	Emergence	Induction	Emergence	Induction	Emergence	Induction	Emergence
EC_50_ (%atm)	0.87	0.75	0.77	0.37	0.86	0.79	0.80	0.31
95% C.I. (%atm)	0.86–0.87	0.73–0.77	0.76–0.78	0.35–0.38	0.85–0.88	0.76–0.82	0.77–0.83	0.29–0.34
Hill slope	−30.3	−8.8	−23.0	−3.8	−22.7	−6.5	−16.9	−4.8
95% C.I. (%atm)	−37.4 to −23.2	−10.5 to −7.1	−28.2 to −17.9	−4.4 to −3.3	−29.8 to −15.7	−8.5 to −4.5	−26.4 to −7.5	−6.0 to −3.6

Top is set to 100% in mice.

### Barrier separating an anesthetized state from wakefulness is decreased in *Drosophila* Shaker potassium channel mutants

**Figure 6:**
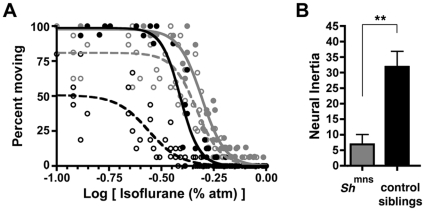
Genetic Changes Also Modulate Neural Inertia In Flies. (**A**) Isoflurane dose-response curve in flies with a mutant *Shaker* potassium channel, *Sh* ^mns^ (gray circles) and sibling controls (black circles) is shown for induction (filled symbols, solid curves) and emergence (open symbols, dotted curves). (**B**) Unpaired t-test demonstrates that neural inertia is significantly reduced in *Sh* ^mns^ flies. X-axis in A corresponds to the log of the inhaled anesthetic concentration. ^**^p<0.01.

Norepinephrine is a potent arousal-promoting stimulus in mammals. To determine if mechanisms that regulate arousal in the fly also affect behavioral state barriers, we tested one such mechanism. In *Drosophila*, the *Shaker* potassium channel (*Sh*) decreases neural activity and promotes sleep. Consequently, loss of function *Sh* mutants show reduced sleep and increased arousal. Such mutants also exhibit resistance to anesthetic induction [27], [28], [29]. We therefore studied flies carrying the minisleep *Shaker* mutant allele (*Sh* ^mns^) to assess their barrier to state changes. Consistent with published results, *Sh* ^mns^ mutants exhibit significant resistance to induction of anesthesia by isoflurane when compared to sibling controls. However, the most striking phenotype of these flies is reduced neural inertia (Figure 6A–B). A significantly greater fraction of *Sh* ^mns^ mutant flies emerge during the course of downward anesthetic titration as compared to their sibling controls. This behavioral change is largely due to a rightward shift in the EC_50_ for emergence, which translates into collapsed hysteresis, and is measured by reduced neural inertia in *Sh* ^mns^ mutants. The *Sh* ^mns^ mutant flies' EC_50_ for emergence exceeds the EC_50_ for induction of their wild type sibling controls (Figure 6A and Table 3).

**Table 3:** Best fit parameters for isoflurane studies in *Drosophila* carrying the *Sh* ^mns^ mutation and their sibling controls.

	*Sh* ^mns^		*Sh* control siblings	
	Induction	Emergence	Induction	Emergence
EC_50_ (%atm)	0.49	0.47	0.39	0.27
95% C.I. (%atm)	0.48–0.51	0.44–0.49	0.38–0.40	0.25–0.30
Hill slope	−7.8	−7.5	−9.6	−5.7
95% C.I. (%atm)	−9.1 to −6.5	−9.7 to −5.3	−12.0 to −6.8	−8.2 to −3.3
Top	98	81	99	51
95% C.I. Top	93–100	76–86	93–100	46–56

## Discussion

Using general anesthetics, we establish the existence of a fundamental and previously unrecognized property of neural circuits to resist state changes in arousal. We demonstrate that hysteresis between the forward and reverse paths through which the state of anesthesia arises and dissipates cannot be explained solely by pharmacokinetics. Rather, we report novel experimental evidence for a first-order phase transition to and from unconscious states [7], [30]. Once a population of individuals undergoes a transition from wakefulness to anesthetic-induced unconsciousness, that population exhibits resistance to the return of the wakeful state. We use the term neural inertia to describe the experimental representation of the behavioral state barrier and propose that it must dissipate prior to anesthetic emergence and normalization of cognitive function. Hysteresis between anesthetic induction and emergence suggests that the neural substrates modulating arousal state exhibit memory. While the identity of these neural substrates remains unknown, the difference in neural inertia between animals anesthetized with isoflurane and halothane is not altogether unexpected [31]. Two anesthetics that have identical molecular and neuronal targets should give rise to identical hysteresis. However, halothane and isoflurane exhibit differences in protein binding, receptor modulation, and as well as in their effects on neuronal circuits hypothesized to regulate wakefulness [32], [33], [34], [35].

Many examples of hysteresis exist in nature. The melting and freezing temperatures for agar are 85°C and 40°C, respectively. Like 60°C agar, a mouse or fly breathing 0.4% halothane might be anesthetized or awake depending upon its previous state of arousal. Another property of systems exhibiting hysteresis is the presence of stable states in equilibrium. Hysteretic processes are buffered against random or small fluctuations that might otherwise precipitate a transition between states. These fortuitous features are advantageous for clinical anesthesia, for once anesthetized, subjects are unlikely to emerge spontaneously.

Our data also reveal a second property that differentiates conscious ablation from its restoration. In wild type mice and flies, the Hill slopes are steeper during anesthetic induction, indicating less population variability from environmental or genetic sources in comparison to anesthetic emergence. These results likely indicate the underlying increased complexity of the conscious state and suggest that it is more difficult to constitute, or reconstitute, a conscious state than it is to disrupt the conscious wakeful state. Hill slopes for emergence in *Dbh* null mice are significantly less than that of their sibling controls, indicating that the loss of an arousal promoting signal increases variability in emergence, perhaps as the remaining loci struggle to reconstitute the wake state. Consistently, Hill slopes for induction and emergence in *Sh* ^mns^ flies are indistinguishable. However as induction Hill slopes only trended to be greater than emergence Hill slopes in *Sh* ^mns^ sibling controls, it is impossible to definitively assign the change in these slopes to the *Sh* ^mns^ allele rather than to another gene in the genetic background of both groups of flies.

The discovery of behavioral state barriers in two distantly related phyla suggests that this property of nervous systems either emerged at least twice independently or that it arose early in evolution predating the split between arthropods and chordates. Either way these barriers are present across phyla and likely exists in humans as well. Despite non-steady-state conditions, clinical evidence of hysteresis upon induction and emergence in humans from general anesthesia is recognized, but has been historically treated as an artifact, and modeled as a smoothed average to obliterate the asymmetry of drug-induced suspension and reanimation of consciousness [12], [13], [14]. Nonetheless, carefully controlled clinical studies to definitively confirm anesthetic hysteresis in steady-state conditions as evidence of a barrier to anesthetic emergence are currently lacking. Evidence of barriers to the return of wakefulness is known in the neurobiology of natural human sleep. Studies show that humans may exhibit variable periods of confusion, disorientation, and low arousal upon awakening. This poorly understood phenomenon has been labeled sleep inertia [36], which illustrates an intrinsic example of naturally occurring resistance to changes in arousal state.

We show by direct measurement that the residual anesthetic in mouse brain at the EC_50_ for induction is always greater than at the EC_50_ for emergence and rebut the idea that measured inspired anesthetic concentrations simply lag behind CNS tissue concentrations. However, the most convincing data to refute a pharmacokinetic explanation come from studies in *Drosophila*. Respiratory physiology in *Drosophila* simplifies the problem of anesthetic uptake and delivery. Flies utilize a pure diffusion-based respiratory system for gas exchange in which air enters directly into their branching tracheal system that courses throughout the entire organism [37]. While air entry and exit in *Drosophila* occurs through sphincter-controlled openings called spiracles, these portals are never fully closed. Although the fly has a primitive circulatory system, it does not affect oxygen transport or carbon dioxide removal [38]. Hence, in contrast to mammals, the fly circulatory system should not affect anesthetic gas delivery. Mathematical modeling based upon the volume of the cylinder housing individual flies and the measured gas flow dictates that anesthetic equilibrium in the chamber, and the fly itself, should be reached in seconds. Direct measurements of residual halothane in whole flies studied under equilibrium conditions also show a greater tissue concentration of halothane at the EC_50_ for induction than at the EC_50_ for emergence. Still, the best evidence against a pharmacokinetic explanation for our data comes from genetic studies of *Sh* ^mns^ flies where a single gene mutation collapses neural inertia without any obvious effects on volatile anesthetic uptake, distribution, or metabolism.

Modulation of neural inertia can occur by simultaneous leftward or rightward shifts in both induction and emergence curves, during which emergence is affected more than induction to widen or narrow the hysteresis loop. These shifts can be induced genetically as in the case of *Dbh* null mice, where absence of adrenergic ligands results in marked increase in neural inertia (Figure 5). This result is consistent with the observation that adrenergic projections to the thalamus, hypothalamus, and basal forebrain play a critical role in the regulation of arousal [39], [40], [41], [42]. One question that arises is whether increased neural inertia in *Dbh* null mice is due to the loss of adrenergic ligands in the periphery, CNS, or both. CNS specific rescue of adrenergic signaling [26] restores the widened neural inertia of *Dbh* null animals back to control levels, whereas vehicle treatment does not. Notably, while CNS specific rescue leaves peripheral tissues such as the heart, vasculature, and lungs devoid of norepinephrine and epinephrine, restoration of adrenergic signaling in brainstem respiratory and/or vasomotor centers could partially confound an otherwise clean rescue. Arguing against this latter indirect effect in the periphery is the observation that L-DOPS restores brainstem levels of epinephrine and norepinephrine less efficiently than in forebrain [26].

Conversely neural inertia can be narrowed by genetic manipulation as demonstrated by the effects of the mutant allele *Sh* ^mns^ in *Drosophila*. This result is consistent with work done in rats, which demonstrated that microinjection of an antibody against the Shaker potassium channel (K_v_1.2) into the central medial thalamus also reverses deep states of anesthetic-induced hypnosis by abruptly triggering emergence, with signs of return to consciousness, despite ongoing delivery of volatile anesthetics [43]. Near-total collapse of neural inertia in Shaker mutant flies raises concerns of additional anesthetic morbidity. Should they exist, humans with similar low levels of neural inertia may be predisposed to awareness under anesthesia.

In humans, case reports document profoundly delayed emergence in a subset of narcoleptic patients without apparent changes in sensitivity to induction of anesthesia [44], [45]. Narcolepsy with cataplexy is caused by a derangement of orexin (also known as hypocretin) signaling [46], [47]. Orexin-deficient mice exhibit normal sensitivity to induction by isoflurane with delayed emergence from anesthesia, pointing to an increase in neural inertia in these animals [48].

Our initial studies have focused upon candidate genes known to affect the regulation of arousal state [49], [50] and suspected to alter induction of general anesthesia [23], [24], [27], [28]. Whether altered propensity to maintain wakefulness is a necessary condition of altered neural inertia remains unknown, but eminently testable in both mouse and fly models. Moreover, an opportunity exists to exploit the power of genetics in *Drosophila* with the explicit purpose of identifying novel genes that affect sensitivity to induction, emergence, and the inertial barrier separating the two. Understanding the genes and neuronal circuits underlying resistance to behavioral state changes will provide greater insights into mechanisms of drug-induced suspension and reassembly of cognition while also shedding light on the minimal neural substrates required for arousal.

## Materials and Methods

### Ethics Statement

All studies were approved by the Institutional Animal Care and Use Committee at the University of Pennsylvania (Philadelphia, PA) #A3079-01 and were conducted in accordance with the National Institutes of Health guidelines.

### Righting Reflex Studies

48 10–12 week old male C57BL6/J mice (Jackson Laboratories, Bar Harbor, ME) were used in this study. Following four days of habituation, during which mice were exposed to 125 ml/min of fresh oxygen flow in 250 ml cylindrical open circuit chambers for 2 hours, anesthetic testing began [51]. Beginning at zeitgeber time (ZT) 0–2, mice were exposed to a single concentration of halothane dissolved in 100% oxygen for 15 minutes before assessment of the righting reflex was made. Initial and final halothane concentrations were 0.65% and 0.93% with 6 intermediate steps averaging 0.035% apiece. After the last mouse had lost its righting reflex, halothane concentration was decreased in twenty-three 15-minute intervals that averaged 0.04% per step. For isoflurane experiments, average initial and final concentrations were 0.67% and 1.02% with 8 intermediate steps averaging 0.035% apiece. To determine emergence in mice, isoflurane concentration was decreased every 15 minutes using eleven 15-minute steps and an average decrease of 0.04%. Anesthetic gas concentrations were determined in triplicate using a Riken FI-21 refractometer. Body temperature was maintained at 36.6±0.2°C by submerging the chambers in a heated water bath. To minimize the number of animals required, behavioral assessment of righting reflex was conducted with a single anesthetic twice in all mice with one week between exposures. Halothane and isoflurane induction-emergence curves were generated from two independent cohorts of 24 mice. Assessment of isoflurane sensitivity was also performed in *Dbh* heterozygous (n = 13) and null siblings (n = 10) that have been maintained on a hybrid C57BL/6J×129/SvCPJ genetic background [49]. *Dbh* heterozygous females were mated to *Dbh* null males and treated with 100 µg/ml each of phenylephrine and isoproterenol (Sigma, St. Louis, MO) from embryonic day 8.5 to 16.5 and with 2 mg/ml L-threo-3,4-dihydroxyphenylserine (L-DOPS, Sumitomo Pharmaceuticals, Osaka, Japan) from E16.5 to birth in the maternal drinking water to enhance fetal survival [52]. As neither norepinephrine nor epinephrine is essential for postnatal survival, litters were not treated after birth. Mice ranged in age from 4–6 months and included equal numbers of males and females.

### Pharmacologic rescue of catecholamine signaling in *Dbh* null mice

Rescue of adrenergic signaling in *Dbh* null mice was performed in accordance with published protocols [26], [49]. Five hours prior to beginning anesthetic sensitivity testing, mice received a subcutaneous injection of 20 mg/ml pH neutralized L-DOPS plus 2 mg/ml vitamin C (Sigma, St. Louis, MO) and 1 mg/ml of the peripheral aromatic L-amino acid decarboxylase inhibitor, benserazide (Sigma, St. Louis, MO), in a final volume of 50 µl/g. Such treatment has been shown to restore near-normal levels for 12 hours with levels peaking roughly 5 hours after injection [26]. To control for the stress of injection, half the animals received a subcutaneous injection of vehicle 50 µl/g 5 hours prior to behavioral testing.

### Behavioral assessment of anesthetic action in *Drosophila*

Fly strains used were *Iso31* (an isogenic *w^1118^* strain) and *RC1*. Flies with the *Sh* ^mns^ mutation were provided by Dr. Chiara Cirelli (University of Wisconsin) and outcrossed six times into an *Iso31* background. Flies were housed under 12∶12 hr Light∶Dark conditions and maintained under standard conditions [53]. Two to three days post eclosion, adult female flies were anesthetized with carbon dioxide and placed into 65mm×5mm cylindrical tubes containing 5% sucrose and 2% agar and entrained for 24 hours. Baseline locomotor/rest activity was then measured for 24 hours using a locomotor monitoring system (Trikinetics, Waltham, MA). Experiments were conducted during the evening locomotor activity peak (ZT10.5 to ZT13) [20], [21]. Anesthetics dissolved in air were delivered to flies in parallel circuit design [51]. Anesthetic gas concentration was measured as described above. To exclude endogenous sleep and/or hypothermia (as continuous gas flows through the DAMS tubes) as sources of inactivity, locomotor activity of non-anesthetized control flies receiving air was simultaneously measured. Flies were exposed to a set of 5-minute stepwise increases followed by decreases in anesthetic concentration. Fresh gas flow was measured with a mass flowmeter (Omega, Stamford, CT) and set at 15 ml/min/tube. Based upon a measured tube volume of 0.75ml, each cylindrical tube housing a single fly should reach equilibrium within 18 seconds. Activity counts were summed over all 5 minutes spent at a single anesthetic concentration. Counts for each individual fly were transformed to a binary output of 0, signifying no activity, or 1, indicating movement. Flies that did not move in the 15 minutes prior to the start of anesthesia or during the first 5 minutes at the lowest anesthetic dose were excluded from subsequent analysis. Flies that did not recover activity during the 24 hours following anesthesia were also excluded from analysis. In total less than 2% of flies were excluded, Table S1.

### Tissue measurements of volatile anesthetic concentration in mouse brain or whole fly

1–2 weeks after the second determination of the population's EC_50_ for induction and emergence, a subset of mice from each original cohort (isoflurane, n = 18; halothane, n = 16) were exposed to an identical anesthetic ramp-up and down protocol for the third time. At the EC_50_ dose for induction, half of the mice were sacrificed while the remaining half continued in the dosing protocol, until all mice had lost their righting reflex. Subsequently, isoflurane or halothane levels were decreased in 15-minute steps until the EC_50_ for emergence was reached when the remaining mice were sacrificed. Upon sacrifice, whole brains were processed as previously described [48]. To determine halothane levels in flies at the EC_50_ doses for induction and emergence, populations of 100 flies were simultaneously exposed to halothane using an identical concentration ramp in the barrel of a 10ml syringe. At the EC_50_ corresponding to induction (5 groups) of anesthesia, flies were snap frozen in liquid nitrogen. A second cohort of flies (4 groups) underwent a full induction and following peak halothane concentration, decreasing doses were delivered. At the EC_50_ for emergence flies were snap frozen in liquid nitrogen. Groups of whole flies were homogenized and measured by HPLC as described as previously described. Unlike mice, whole fly homogenates cannot be assumed to yield central concentrations.

### Statistical analyses

Induction and emergence curves were fit with a sigmoidal dose-response and variable slope function (Prism 4.0c, GraphPad Software Inc., San Diego, CA) as described [48], [51]. Each curve depicts the best-fit data for two to four replicates (mice) or three to six replicates (*Drosophila*) of the corresponding wild type or mutant populations. For murine studies, “bottom” and “top” parameters were constrained to 0 and 100 respectively. In *Drosophila* studies, “bottom” was constrained to 0, while the “top” was not constrained. No constraints were placed on the Hill slope, EC_50_, or log(EC_50_) fit parameters. All values are reported along with their corresponding 95% confidence intervals. To calculate neural inertia, both induction and emergence sigmoidal dose-response curves were mathematically integrated over the range of the induction curve's EC_1_ to the emergence curve's EC_99_ corresponding to the concentrations at which 99% of the population had entered or exited from the anesthetic state. Neural inertia for each set of induction and emergence curves is expressed as the mean ± standard error. (Appendix S1) Comparison of neural inertia between wild type and mutants is reported using a t-test or one-way ANOVA as appropriate. Anesthetic concentrations in whole fly or mouse brain are reported as the average ± standard error with significance determined by t-tests.

## Supporting Information

Table S1 Number of Study Subjects.
(0.03 MB DOC)

Figure S1 Graphical Depictions Of Neural Inertia Arising With Different Hill Slope, LogEC50, And Top Best-Fit Parameters. Neural inertia is shown in red and defined by the area bounded between the induction and emergence curves over the X-range corresponding to the emergence EC99 (denoted by the dashed vertical line labeled E99) through the induction EC1 (denoted by the solid vertical line labeled I1). Due to hysteresis that separates the induction and emergence curves, the E99≠I99 and the E1≠I1.
(0.31 MB TIF)

Figure S2 Neural Inertia In Wild Type RC1 Drosophila Strain. RC1 flies have the wild type w gene allele. Filled circles and their corresponding best-fit solid curve denote induction. Open circles and their corresponding best-fit dashed curve denote emergence. (A) Isoflurane induction and emergence dose-response curves in RC1 flies. (B) Halothane induction and emergence dose-response curves in RC1 flies. (C) Neural inertia in RC1 flies.
(0.77 MB TIF)

Appendix S1 Derivation of Neural Inertia.
(0.06 MB DOC)

## References

[1] Fort P, Bassetti CL, Luppi PH (2009). Alternating vigilance states: New insights regarding neuronal networks and mechanisms.. Eur J Neurosci.

[2] Saper CB, Chou TC, Scammell TE (2001). The sleep switch: Hypothalamic control of sleep and wakefulness.. Trends Neurosci.

[3] Chatterjee A, Kaznessis YN, Hu WS (2008). Tweaking biological switches through a better understanding of bistability behavior.. Curr Opin Biotechnol.

[4] Jonson B, Svantesson C (1999). Elastic pressure-volume curves: What information do they convey?. Thorax.

[5] Sethna JP, Dahmen K, Kartha S, Krumhansl JA, Roberts BW (1993). Hysteresis and hierarchies: Dynamics of disorder-driven first-order phase transformations.. Phys Rev Lett.

[6] Shadwick RE (1990). Elastic energy storage in tendons: Mechanical differences related to function and age.. J Appl Physiol.

[7] Steyn-Ross ML, Steyn-Ross DA, Sleigh JW (2004). Modelling general anaesthesia as a first-order phase transition in the cortex.. Prog Biophys Mol Biol.

[8] Rudolph U, Antkowiak B (2004). Molecular and neuronal substrates for general anaesthetics.. Nat Rev Neurosci.

[9] Alkire MT, Hudetz AG, Tononi G (2008). Consciousness and anesthesia.. Science.

[10] Franks NP (2008). General anaesthesia: From molecular targets to neuronal pathways of sleep and arousal.. Nat Rev Neurosci.

[11] Lydic R, Baghdoyan HA (2005). Sleep, anesthesiology, and the neurobiology of arousal state control.. Anesthesiology.

[12] Bruhn J, Ropcke H, Rehberg B, Bouillon T, Hoeft A (2000). Electroencephalogram approximate entropy correctly classifies the occurrence of burst suppression pattern as increasing anesthetic drug effect.. Anesthesiology.

[13] Kreuer S, Wilhelm W, Grundmann U, Larsen R, Bruhn J (2004). Narcotrend index versus bispectral index as electroencephalogram measures of anesthetic drug effect during propofol anesthesia.. Anesth Analg.

[14] McKay ID, Voss LJ, Sleigh JW, Barnard JP, Johannsen EK (2006). Pharmacokinetic-pharmacodynamic modeling the hypnotic effect of sevoflurane using the spectral entropy of the electroencephalogram.. Anesth Analg.

[15] Laureys S (2005). The neural correlate of (un)awareness: Lessons from the vegetative state.. Trends Cogn Sci.

[16] Allada R, Nash HA (1993). Drosophila melanogaster as a model for study of general anesthesia: The quantitative response to clinical anesthetics and alkanes.. Anesth Analg.

[17] Gamo S (2002). Studies on target genes of general anesthetics.. Curr Drug Targets.

[18] van Swinderen B (2006). A succession of anesthetic endpoints in the Drosophila brain.. J Neurobiol.

[19] van Swinderen B, Andretic R (2003). Arousal in Drosophila.. Behav Processes.

[20] Shaw PJ, Cirelli C, Greenspan RJ, Tononi G (2000). Correlates of sleep and waking in Drosophila melanogaster.. Science.

[21] Hendricks JC, Finn SM, Panckeri KA, Chavkin J, Williams JA (2000). Rest in Drosophila is a sleep-like state.. Neuron.

[22] Campbell JL, Nash HA (2001). Volatile general anesthetics reveal a neurobiological role for the white and brown genes of Drosophila melanogaster.. J Neurobiol.

[23] Miller RD, Way WL, Eger EI, 2nd (1968). The effects of alpha-methyldopa, reserpine, guanethidine, and iproniazid on minimum alveolar anesthetic requirement (MAC).. Anesthesiology.

[24] Mueller RA, Smith RD, Spruill WA, Breese GR (1975). Central monaminergic neuronal effects on minimum alveolar concentrations (MAC) of halothane and cyclopropane in rats.. Anesthesiology.

[25] Roizen MF, White PF, Eger EI, Brownstein M (1978). Effects of ablation of serotonin or norepinephrine brain-stem areas on halothane and cyclopropane MACs in rats.. Anesthesiology.

[26] Thomas SA, Marck BT, Palmiter RD, Matsumoto AM (1998). Restoration of norepinephrine and reversal of phenotypes in mice lacking dopamine beta-hydroxylase.. J Neurochem.

[27] Tinklenberg JA, Segal IS, Guo TZ, Maze M (1991). Analysis of anesthetic action on the potassium channels of the Shaker mutant of Drosophila.. Ann N Y Acad Sci.

[28] Weber B, Schaper C, Bushey D, Rohlfs M, Steinfath M (2009). Increased Volatile Anesthetic Requirement in Short-sleeping Drosophila Mutants.. Anesthesiology.

[29] Walcourt A, Scott RL, Nash HA (2001). Blockage of one class of potassium channel alters the effectiveness of halothane in a brain circuit of Drosophila.. Anesth Analg.

[30] Steyn-Ross DA, Steyn-Ross ML, Wilcocks LC, Sleigh JW (2001). Toward a theory of the general-anesthetic-induced phase transition of the cerebral cortex. II. Numerical simulations, spectral entropy, and correlation times.. Phys Rev E Stat Nonlin Soft Matter Phys.

[31] Campbell JL, Gu Q, Guo D, Nash HA (2009). Genetic Effects in Drosophila on the Potency of Diverse General Anesthetics: A Distinctive Pattern of Altered Sensitivity.. J Neurogenet.

[32] Correa AM (1998). Gating kinetics of Shaker K+ channels are differentially modified by general anesthetics.. Am J Physiol.

[33] Eckenhoff MF, Eckenhoff RG (1998). Quantitative autoradiography of halothane binding in rat brain.. J Pharmacol Exp Ther.

[34] Gompf HS, Chen J, Sun Y, Yanagisawa M, Aston-Jones G (2009). Halothane-induced Hypnosis is not Accompanied by Inactivation of Orexinergic Output in Rodents.. Anesthesiology.

[35] Keifer JC, Baghdoyan HA, Lydic R (1996). Pontine cholinergic mechanisms modulate the cortical electroencephalographic spindles of halothane anesthesia.. Anesthesiology.

[36] Tassi P, Muzet A (2000). Sleep inertia.. Sleep Med Rev.

[37] Uv A, Cantera R, Samakovlis C (2003). Drosophila tracheal morphogenesis: Intricate cellular solutions to basic plumbing problems.. Trends Cell Biol.

[38] Lighton JR (2005). Respiratory physiology: Strange cycles and the fruit-fly's tongue.. Curr Biol.

[39] McCormick DA, Pape HC, Williamson A (1991). Actions of norepinephrine in the cerebral cortex and thalamus: Implications for function of the central noradrenergic system.. Prog Brain Res.

[40] Gallopin T, Fort P, Eggermann E, Cauli B, Luppi PH (2000). Identification of sleep-promoting neurons in vitro.. Nature.

[41] Berridge CW, Foote SL (1996). Enhancement of behavioral and electroencephalographic indices of waking following stimulation of noradrenergic beta-receptors within the medial septal region of the basal forebrain.. J Neurosci.

[42] Berridge CW, O'Neill J (2001). Differential sensitivity to the wake-promoting actions of norepinephrine within the medial preoptic area and the substantia innominata.. Behav Neurosci.

[43] Alkire MT, Asher CD, Franciscus AM, Hahn EL (2009). Thalamic microinfusion of antibody to a voltage-gated potassium channel restores consciousness during anesthesia.. Anesthesiology.

[44] Mesa A, Diaz AP, Frosth M (2000). Narcolepsy and anesthesia.. Anesthesiology.

[45] Burrow B, Burkle C, Warner DO, Chini EN (2005). Postoperative outcome of patients with narcolepsy. A retrospective analysis.. J Clin Anesth.

[46] Ohno K, Sakurai T (2008). Orexin neuronal circuitry: Role in the regulation of sleep and wakefulness.. Front Neuroendocrinol.

[47] Siegel JM (1999). Narcolepsy: A key role for hypocretins (orexins).. Cell.

[48] Kelz MB, Sun Y, Chen J, Cheng Meng Q, Moore JT (2008). An essential role for orexins in emergence from general anesthesia.. Proc Natl Acad Sci U S A.

[49] Ouyang M, Hellman K, Abel T, Thomas SA (2004). Adrenergic signaling plays a critical role in the maintenance of waking and in the regulation of REM sleep.. J Neurophysiol.

[50] Cirelli C, Bushey D, Hill S, Huber R, Kreber R (2005). Reduced sleep in Drosophila Shaker mutants.. Nature.

[51] Sun Y, Chen J, Pruckmayr G, Baumgardner JE, Eckmann DM (2006). High throughput modular chambers for rapid evaluation of anesthetic sensitivity.. BMC Anesthesiology.

[52] Thomas SA, Matsumoto AM, Palmiter RD (1995). Noradrenaline is essential for mouse fetal development.. Nature.

[53] Joiner WJ, Crocker A, White BH, Sehgal A (2006). Sleep in Drosophila is regulated by adult mushroom bodies.. Nature.

