# Supplementary material for: A Conserved Behavioral State Barrier Impedes Transitions between Anesthetic-Induced Unconsciousness and Wakefulness: Evidence for Neural Inertia
Source: PLoS One. 2010 Jul 30;5(7):e11903. doi: 10.1371/journal.pone.0011903 (PMC2912772; doi:10.1371/journal.pone.0011903)
Supplement: Table S1 — Number of Study Subjects. (0.03 MB DOC) [file pone.0011903.s001.doc]

| Experimental Group | Number of Excluded Subjects | Total Number of Subjects |
| --- | --- | --- |
| *Iso31* flies | 3 | 112 |
| *RC1* flies | 4 | 62 |
| *Sh*^mns^ flies | 0 | 108 |
| *Sh*^mns^ sibling control flies | 0 | 95 |
| C57BL/6J mice | 0 | 48 |
| DBH heterozygotes mice | 0 | 13 |
| DBH null mice | 0 | 10 |

**SI Table 1.** Number Of Study Subjects. Flies not moving in the 15 minutes preceding anesthetic exposure or in the first 5 minutes of the lowest anesthetic dose were excluded from analysis.
