# Supplementary figures and images for: A Conserved Behavioral State Barrier Impedes Transitions between Anesthetic-Induced Unconsciousness and Wakefulness: Evidence for Neural Inertia
Source: PLoS One. 2010 Jul 30;5(7):e11903. doi: 10.1371/journal.pone.0011903 (PMC2912772; doi:10.1371/journal.pone.0011903)

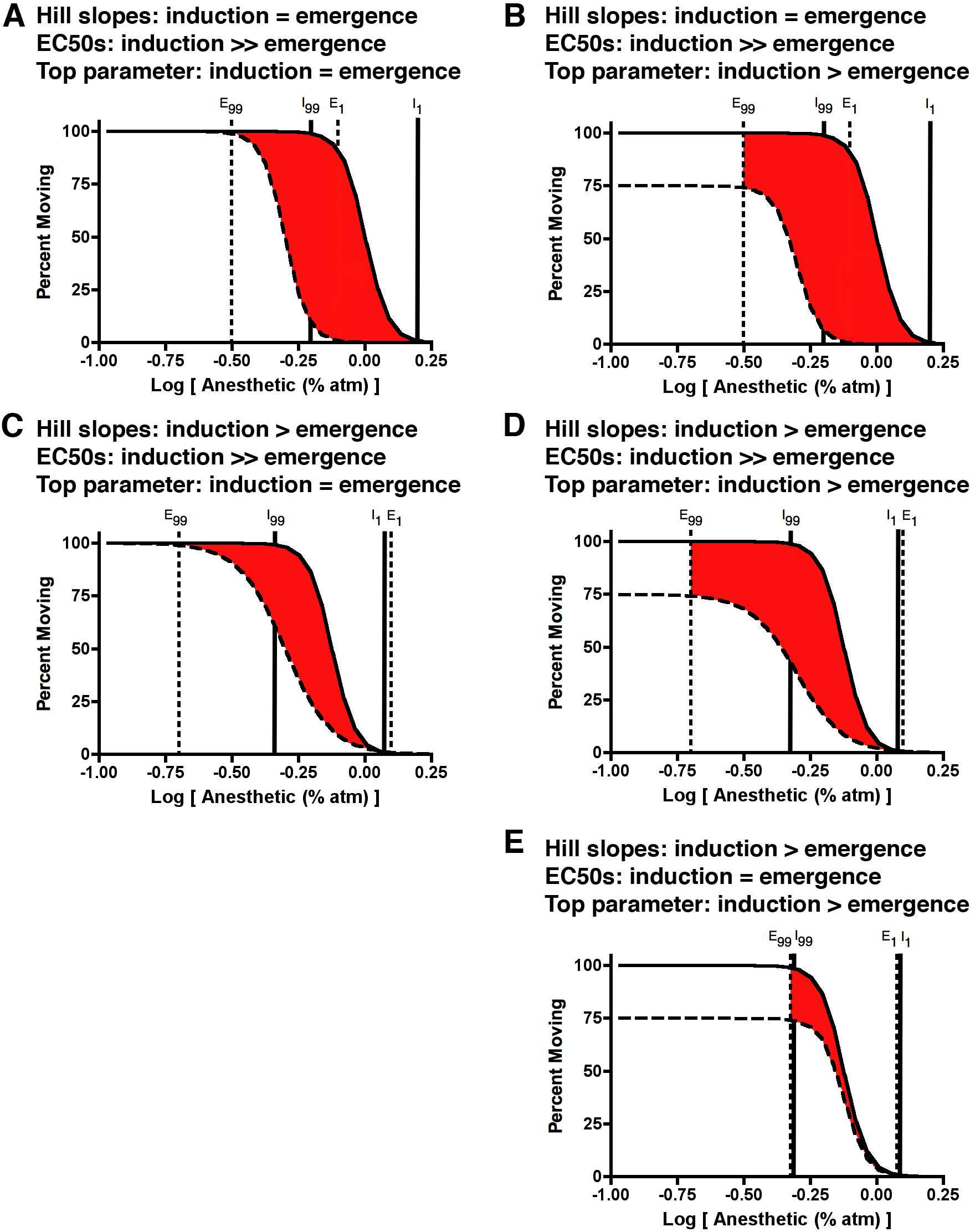

Supplement: Figure S1 — Graphical Depictions Of Neural Inertia Arising With Different Hill Slope, LogEC50, And Top Best-Fit Parameters. Neural inertia is shown in red and defined by the area bounded between the induction and emergence curves over the X-range corresponding to the emergence EC99 (denoted by the dashed vertical line labeled E99) through the induction EC1 (denoted by the solid vertical line labeled I1). Due to hysteresis that separates the induction and emergence curves, the E99≠I99 and the E1≠I1. (0.31 MB TIF) [file pone.0011903.s002.tif]

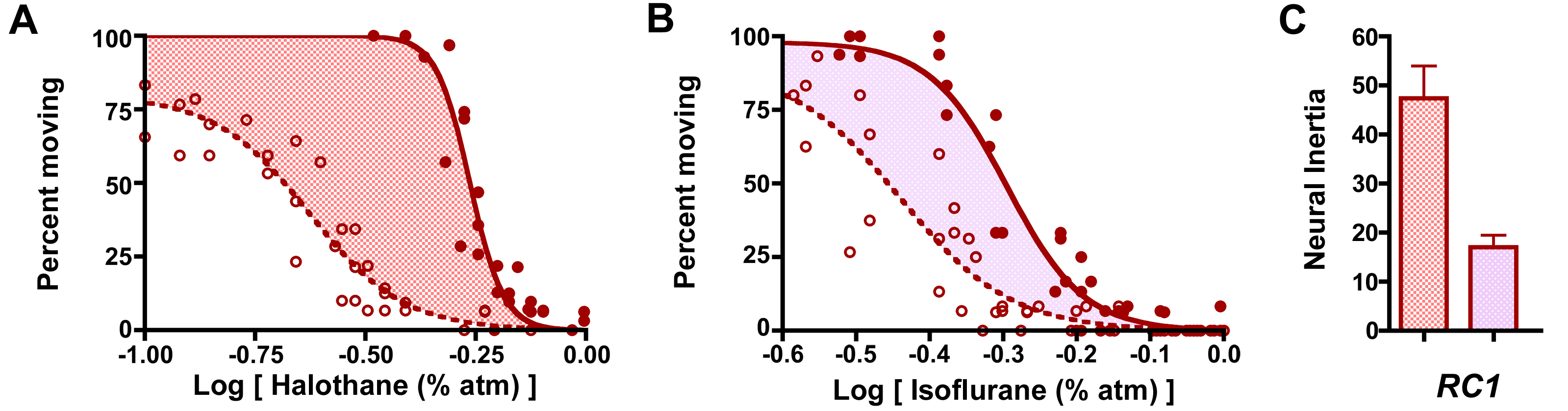

Supplement: Figure S2 — Neural Inertia In Wild Type RC1 Drosophila Strain. RC1 flies have the wild type w gene allele. Filled circles and their corresponding best-fit solid curve denote induction. Open circles and their corresponding best-fit dashed curve denote emergence. (A) Isoflurane induction and emergence dose-response curves in RC1 flies. (B) Halothane induction and emergence dose-response curves in RC1 flies. (C) Neural inertia in RC1 flies. (0.77 MB TIF) [file pone.0011903.s003.tif]
