# Supplementary material for: A Conserved Behavioral State Barrier Impedes Transitions between Anesthetic-Induced Unconsciousness and Wakefulness: Evidence for Neural Inertia
Source: PLoS One. 2010 Jul 30;5(7):e11903. doi: 10.1371/journal.pone.0011903 (PMC2912772; doi:10.1371/journal.pone.0011903)
Supplement: Appendix S1 — Derivation of Neural Inertia. (0.06 MB DOC) [file pone.0011903.s004.doc]

**Appendix S1: Derivation of neural inertia**

Based upon the data in wild type *Iso*31 flies, which don’t all emerge by the end of the experiment, we define neural inertia as the area between the induction and emergence curves corresponding to the x-values of the induction curve’s EC_1_ through the emergence curve’s EC_99_ and shown schematically by the red shaded area in Figure S1. The anesthetic doses corresponding to the emergence EC_99_ and induction EC_1_ are defined respectively by the concentration at which 99% of the population has exited the state of anesthesia (dashed emergence curve) and 1% of the population has not yet entered (solid induction curve). When the top asymptote is less than 100, for example 75 as shown in Fig S1B, S1D, and S1E, then the EC_99_ corresponds to the value that is 99% of the difference between the top and bottom values (or the x-values corresponding to 74.25% of flies still moving) while the EC_1_ corresponds to the value that is 1% of the difference between the top and bottom asymptotes (x-value corresponding to the 0.75% of flies still moving). Note that the x-values corresponding to the induction curve’s EC_1_ and EC_99_ (solid vertical lines denoted by I_1_ and I_99_) do not necessarily equal the respective x-values corresponding to the emergence curve’s EC_1_ and EC_99_ (dashed vertical lines denoted by E_1_ and E_99_).

The necessity for a definite integral was confirmed in another *Drosophila* strain, *RC1* that also fail to fully emerge during the course of the anesthetic exposure. Nevertheless, these slowly emerging flies aren’t dead (Figure 2E,F). The definite integral approach places an upper limit on neural inertia in situations where the top parameter, T, is less during the return to wakefulness (emergence) than it was prior to induction of anesthesia. The mathematical calculation of neural inertia is shown on the pages that follow.

**Mathematical derivation of neural inertia:**

Now if terms EC_50i_, H_i_, T_i_, B_i_ correspond to the best fit values for induction and the terms EC50_e_, H_e_, T_e_, and B_e_ correspond to the best fit values for emergence then:

The range of EC_1i_ through EC_99e_ is admittedly arbitrary as an EC_5i_ to EC_95e_ could also have been a viable alternative. However, we have chosen the EC_1i_ to EC_99e_ range to capture a large slice of the area between the induction and emergence curves to maintain its sensitivity to all of the best-fit constants without being overly weighted to differences in the top constant, T. Recall that at the x value corresponding to the EC_50_ for induction (EC_50i_) or emergence (EC_50e_) the Y value will be or , respectively. These values will be less than 50 if the best-fit top parameter is less than 100.
